# Generative Adversarial Networks for Intrusion Detection Systems: A Comprehensive Survey of Applications, Challenges, and Research Directions

**DOI:** 10.1007/s13369-026-11103-6

**Published:** 2026-02-06

**Authors:** Mohammad Alauthman, Nauman Aslam, Ahmad Al-Qerem, Amjad Aldweesh, Pradorn Sureephong

**Affiliations:** 1 Department of Information Security, University of Petra, Amman, Jordan; 2 School of Computer Science, Northumbria University, Newcastle upon Tyne, UK; 3 Computer Science Department, Zarqa University, Zarqa, Jordan; 4 College of Computer Science and IT, Shaqra University, Riyadh, Saudi Arabia; 5 College of Arts, Media and Technology, Chiang Mai University, Chiang Mai, Thailand

**Keywords:** Generative adversarial networks, Intrusion detection systems, Network security, Adversarial learning, Cybersecurity, Anomaly detection, Synthetic data generation

## Abstract

The evolving threat landscape demands intrusion detection systems that adapt quickly to novel attack patterns and operate across heterogeneous environments. Recent studies show that Generative Adversarial Networks (GANs) can improve intrusion detection performance by generating synthetic attack traffic, balancing imbalanced datasets, enhancing adversarial robustness, and serving as anomaly detectors. This survey provides a comprehensive and systematic review of GAN-based intrusion detection system (IDS) research, analyzing the architectures employed—including Wasserstein GANs, conditional GANs, self-attention GANs, and specialized multi-generator designs—together with their applications, datasets, and evaluation metrics. Unlike previous surveys, we extend the scope to resource-constrained Internet of Things (IoT) and federated scenarios, where lightweight and tabular GANs can process sensor data and operate on edge devices. We also examine deployments in software-defined networking environments. We propose a unified evaluation framework that reports class-wise precision, recall and macro-F1-scores, per-attack metrics, computational cost, and statistical similarity tests, and we emphasize the need for interpretable and multi-modal approaches that fuse network flows with logs or threat intelligence. Emerging paradigms including GANs combined with large language models, quantum GANs, diffusion models, and reinforcement learning are surveyed, and open challenges such as training instability, mode collapse, hyper-parameter tuning, and ethical dual-use concerns are discussed. By synthesizing recent advances and outlining future research directions, this survey provides a comprehensive and forward-looking reference for practitioners and researchers developing robust, privacy-preserving, and adaptive GAN-based intrusion detection systems.

## Introduction

### Background and Motivation

Cybersecurity threats continue to grow in sophistication and frequency, with organizations facing increasingly complex attack vectors. Traditional rule-based intrusion detection systems (IDS) often struggle to detect zero-day attacks due to their reliance on known signatures or patterns [1]. This limitation has driven research toward more adaptive and intelligent detection mechanisms, particularly those leveraging machine learning and artificial intelligence (AI).

**Fig. 1:**
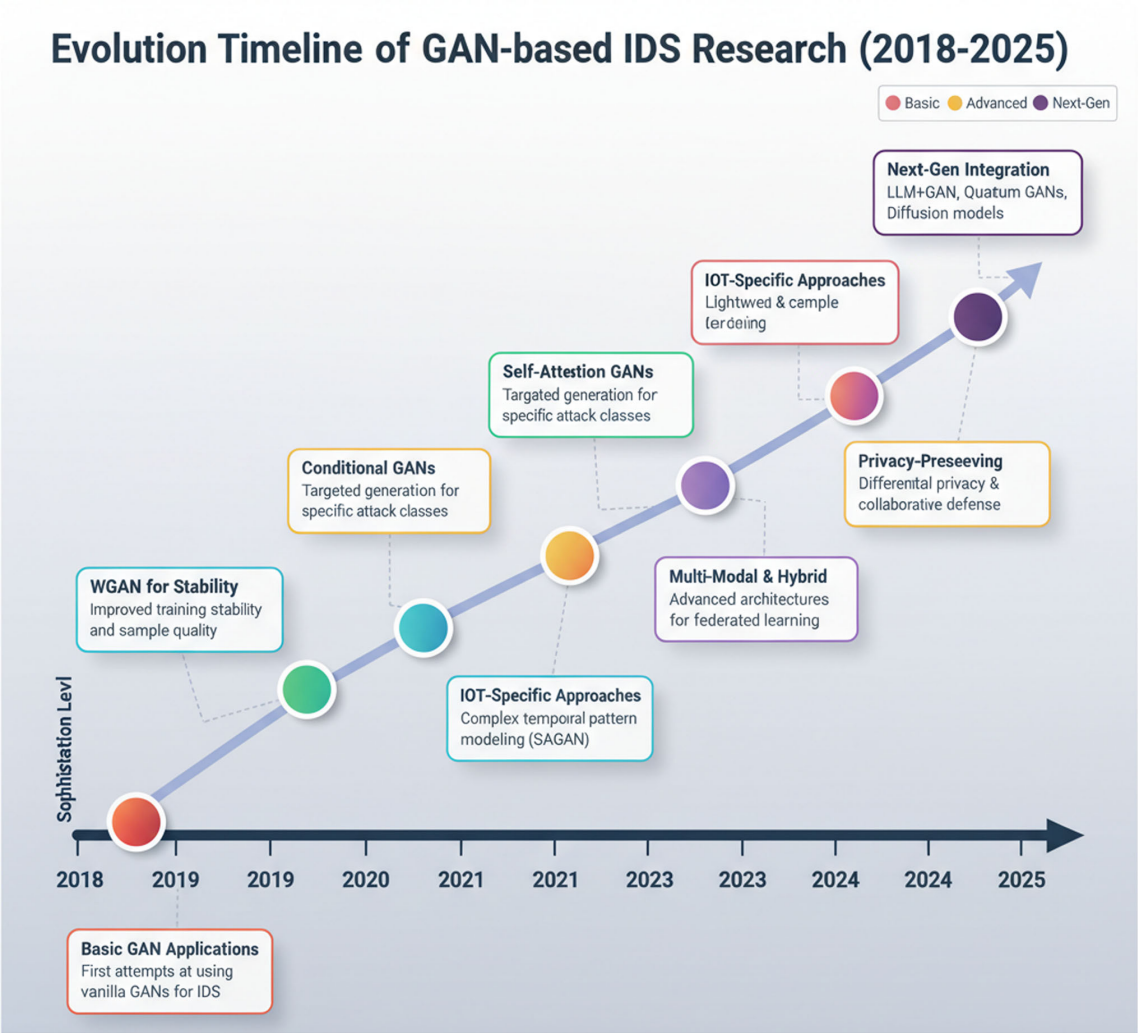
Evolution timeline of GAN-based intrusion detection research from 2018 to 2025, showing the progression from basic applications to sophisticated architectures addressing modern cybersecurity challenges including IoT security and privacy preservation

Since Goodfellow et al. [2] introduced Generative Adversarial Networks (GANs) in 2014, they have transformed many fields by enabling the generation of synthetic data that closely match real-world data distributions. In cybersecurity, GANs are intrinsically dual use: they can strengthen defenses—for example, by augmenting imbalanced intrusion detection system (IDS) datasets [3–5]—but they can also support offensive aims, such as generating evasive or adversarial traffic intended to bypass detection [6–8]. As shown in Fig. 1, research on GAN-based intrusion detection has progressed from early, relatively simple implementations to increasingly sophisticated architectures designed to address contemporary cybersecurity threats.

The use of Generative Adversarial Networks (GANs) in intrusion detection has its own opportunities and challenges. On the one hand, GANs can be used to generate synthetic attack traffic, which can be included in training sets, eliminating the imbalance of classes and improving the performance of detectors with little encountered attack modalities in the training sets [5, 9]. On the other hand, the opponents can use GANs to generate evasive attack traffic to evade detection systems [7, 8]. Such duality creates the spirit of competition between defenders and attackers, therefore leading to the constant innovation in the sphere of cybersecurity research.

Over the past several years, GAN architectures and their implementation on intrusion detection have experienced numerous improvements. The variants adapted to network traffic and security data and data-specific needs are specifically designed with Wasserstein GANs (WGANs), Conditional GANs (CGANs), and Self-Attention GANs, which have been developed to meet particular network traffic and security data needs of these domains individually [1, 5]. As a consequence, these innovations have increased detection, lowered incidences of false positives, and increased resistance to adversarial attacks.

### Research Objectives

This comprehensive survey aims to analyze the current state of research on GANs for intrusion detection systems, with particular emphasis on recent developments. Specifically, we seek to address the following research questions:

1. **RQ1:** What GAN architectures are predominantly used in intrusion detection systems, and what are their relative strengths and limitations?
2. **RQ2:** How are GANs being applied to enhance different aspects of intrusion detection, including data augmentation, adversarial training, and anomaly detection?
3. **RQ3:** Which datasets and evaluation metrics are commonly used to assess GAN-based intrusion detection systems?
4. **RQ4:** What are the key challenges, research gaps, and promising future directions in this field?
5. **RQ5:** How does the dual-use nature of GANs (for both attack and defense) impact the development of intrusion detection systems?

### Survey Scope and Organization

These questions (Sect. 1.2) guide our systematic examination of the literature and inform our analysis of current trends, gaps, and future directions in the field. We include studies that apply GANs to network-based intrusion detection systems (NIDS), host-based intrusion detection systems (HIDS), and hybrid approaches. We focus on intrusion detection in network-based, host-based, IoT, and software-defined networking (SDN) environments and include only studies in which GANs are explicitly evaluated as part of an intrusion detection or closely related attack detection task.

Several surveys have recently reviewed the use of GANs for cybersecurity, including work focused on malware detection [10] and a 2024 review of GAN-based IDS techniques by Al-Ajlan and Ykhlef [11]. Compared with these studies, our survey (i) covers more recent work up to 2025, (ii) places particular emphasis on IoT, federated and SDN-based IDS deployments, (iii) systematically analyzes privacy-preserving and diffusion/quantum generative models that are only briefly mentioned in earlier reviews, and (iv) proposes a unified evaluation framework that stresses class-wise metrics, per-attack analysis and computational cost. This positioning clarifies how our contribution complements and extends prior surveys.

The remainder of this paper is organized as follows: Sect. 2 describes the survey methodology. Section 3 reviews GAN and IDS fundamentals. Section 4 analyzes GAN architectures for IDS. Section 5 discusses GAN applications in IDS. Section 6 analyzes datasets, metrics, and performance benchmarks. Section 7 outlines open challenges and future research directions. Finally, Sect. 8 concludes with key insights and recommendations.

## Methodology

### Search Strategy and Study Selection

In this survey, a systematic literature search was conducted in digital libraries and scholarly databases to find the studies that are applicable to the application of generative adversarial networks in intrusion detection. The search was made in leading repositories such as IEEE Xplore, the ACM Digital Library, SpringerLink, ScienceDirect, arXiv, and Google Scholar. The search string used was as follows with relevant changes to each database:

(“generative adversarial network” OR “GAN” OR “WGAN” OR “CGAN”) AND (“intrusion detection” OR “network security” OR “anomaly detection” OR “cyber security” OR “threat detection”)

The initial search yielded potentially relevant studies. After removing duplicates, unique publications remained for screening.

- Studies that apply GANs specifically to intrusion detection problems
- Peer-reviewed journal articles, conference papers, or substantive preprints
- Publications in English

We applied the following exclusion criteria:

- Studies focusing solely on GAN theory without application to intrusion detection
- Short papers, extended abstracts, or presentations without substantial technical content
- Studies with insufficient detail on GAN architecture, methodology, or results

### Data Extraction and Synthesis

From each included study, we extracted the following information:

- Study metadata (authors, year, publication venue)
- GAN architecture and implementation details
- Detection approach (anomaly-based, signature-based, hybrid)
- Network type (NIDS, HIDS, hybrid)
- Attack types detected
- Datasets used
- Evaluation metrics and performance results
- Feature selection methods
- Key findings and limitations
- Future research directions

## Background

### Generative Adversarial Networks

In a GAN, a generator learns to produce synthetic samples that mimic the training data distribution, while a discriminator learns to distinguish real samples from generated ones. Training proceeds as an adversarial minimax game until the discriminator can no longer reliably distinguish generated samples from authentic observations.

The original GAN formulation can be expressed as a minimax game:

1 $$\begin{aligned} {\begin{matrix} \min _{G} \max _{D} V(D,G) & = \mathbb {E}_{x \sim p_{data}(x)}[\log D(x)] \\ & \quad + \mathbb {E}_{z \sim p_{z}(z)}[\log (1 - D(G(z)))] \end{matrix}} \end{aligned}$$

where

- *G* represents the generator
- *D* represents the discriminator
- $p_{data}(x)$ is the distribution of real data
- $p_{z}(z)$ is a prior distribution (typically Gaussian) from which the generator draws input
- *G*(*z*) is the generated data sample
- *D*(*x*) is the discriminator’s estimate of the probability that *x* is real

Since their introduction, numerous GAN variants have been developed to address challenges such as training instability, mode collapse, and application-specific requirements. Key variants relevant to intrusion detection include:

- **Wasserstein GAN (WGAN)** [5, 12]: Replaces the original GAN’s Jensen–Shannon divergence-based objective with the Earth Mover’s (Wasserstein) distance, improving training stability and mitigating mode collapse.
- **Conditional GAN (CGAN)** [4, 13, 14]: Conditions both the generator and discriminator on auxiliary information (e.g., class labels) to enable class-specific sample generation and more controlled data synthesis.
- **Deep Convolutional GAN (DCGAN)** [15, 16]: Introduces convolutional architectures for both generator and discriminator, learning hierarchical representations that are useful when traffic/features are structured.
- **Self-Attention GAN (SAGAN)** [1, 7, 17]: Incorporates self-attention to capture long-range dependencies, improving modeling of complex traffic patterns and adversarial flows.
- **Auxiliary Classifier GAN (ACGAN)** [18, 19]: Adds an auxiliary classifier to the discriminator to encourage class-conditional generation and preserve discriminative features for minority classes.
- **Tabular GAN (CTGAN)** [5, 20, 21]: Tailors GAN training to mixed continuous/categorical tabular data via conditional sampling and mode-specific normalization, which fits feature-based IDS datasets.

These architectural innovations have significantly enhanced the applicability of GANs to intrusion detection challenges, as we will explore throughout this review.

### Intrusion Detection Systems

An intrusion detection system (IDS) is a core security component that continuously monitors network traffic and host activities. It raises alerts when anomalous behavior or violations of defined security policies are detected. IDSs are typically deployed as complementary controls alongside other mechanisms such as firewalls.

- **Network-based IDS (NIDS)**: Monitors network traffic for suspicious activities or policy violations. NIDS typically analyze packet headers and payloads to identify patterns indicative of attacks such as port scans, denial of service, or protocol anomalies.
- **Host-based IDS (HIDS)**: Monitors the internal activities of a host system, including file access, process behavior, system call patterns, and log files to detect unauthorized or anomalous activities.

IDS can also be categorized based on their detection methodology:

- **Signature-based detection**: Identifies attacks by matching observed activities against pre-defined patterns or signatures of known attacks. While effective for known threats, this approach struggles with zero-day or previously unseen attacks.
- **Anomaly-based detection**: Establishes a baseline of normal system or network behavior and flags deviations from this baseline as potential intrusions. This approach can detect novel attacks but may generate false positives when legitimate activities deviate from the norm.
- **Hybrid detection**: Combines signature-based and anomaly-based approaches to leverage the strengths of both methods.

Machine learning has increasingly been applied to enhance IDS capabilities, particularly for anomaly detection. Traditional ML approaches include decision trees, support vector machines, k-nearest neighbors, and various ensemble methods. Deep learning techniques such as recurrent neural networks (RNNs), convolutional neural networks (CNNs), and autoencoders have demonstrated strong performance in recent years [1, 5].

The application of GANs to intrusion detection represents a significant advancement in this evolution, offering novel approaches to address persistent challenges such as class imbalance, adversarial robustness, and zero-day attack detection [1, 5, 7].

### Challenges in Intrusion Detection

Despite advances in intrusion detection technologies, several challenges persist that motivate the application of GANs:

- **Class Imbalance**: In real-world network environments, malicious traffic typically constitutes a small fraction of overall traffic. This imbalance can bias ML models toward the majority class (benign traffic), resulting in poor detection of attack instances [4, 22].
- **Data Scarcity**: Obtaining comprehensive, labeled datasets for certain attack types (e.g., advanced persistent threats, zero-day exploits) is difficult. This scarcity hampers model training and evaluation [5, 23].
- **Evolving Threat Landscape**: Attackers continuously adapt their techniques to evade detection, requiring IDS to evolve accordingly [5, 7].
- **Adversarial Attacks**: ML-based IDS are vulnerable to adversarial examples—specifically crafted inputs designed to cause misclassification [7].
- **High False Positive Rates**: Many IDS struggle with false alarms, which can lead to alert fatigue and missed genuine attacks [1, 4].
- **Computational Efficiency**: Real-time detection requirements demand models that are both accurate and computationally efficient [1].

GANs offer potential solutions to many of these challenges, as we will explore in the following sections. Their ability to generate synthetic data can address class imbalance and data scarcity, while adversarial training can improve robustness against evasion attempts. However, GANs also introduce new challenges, including training instability, computational demands, and the potential for misuse [10].

## GAN Architectures for Intrusion Detection

### Overview of GAN Variants in IDS

**Fig. 2:**
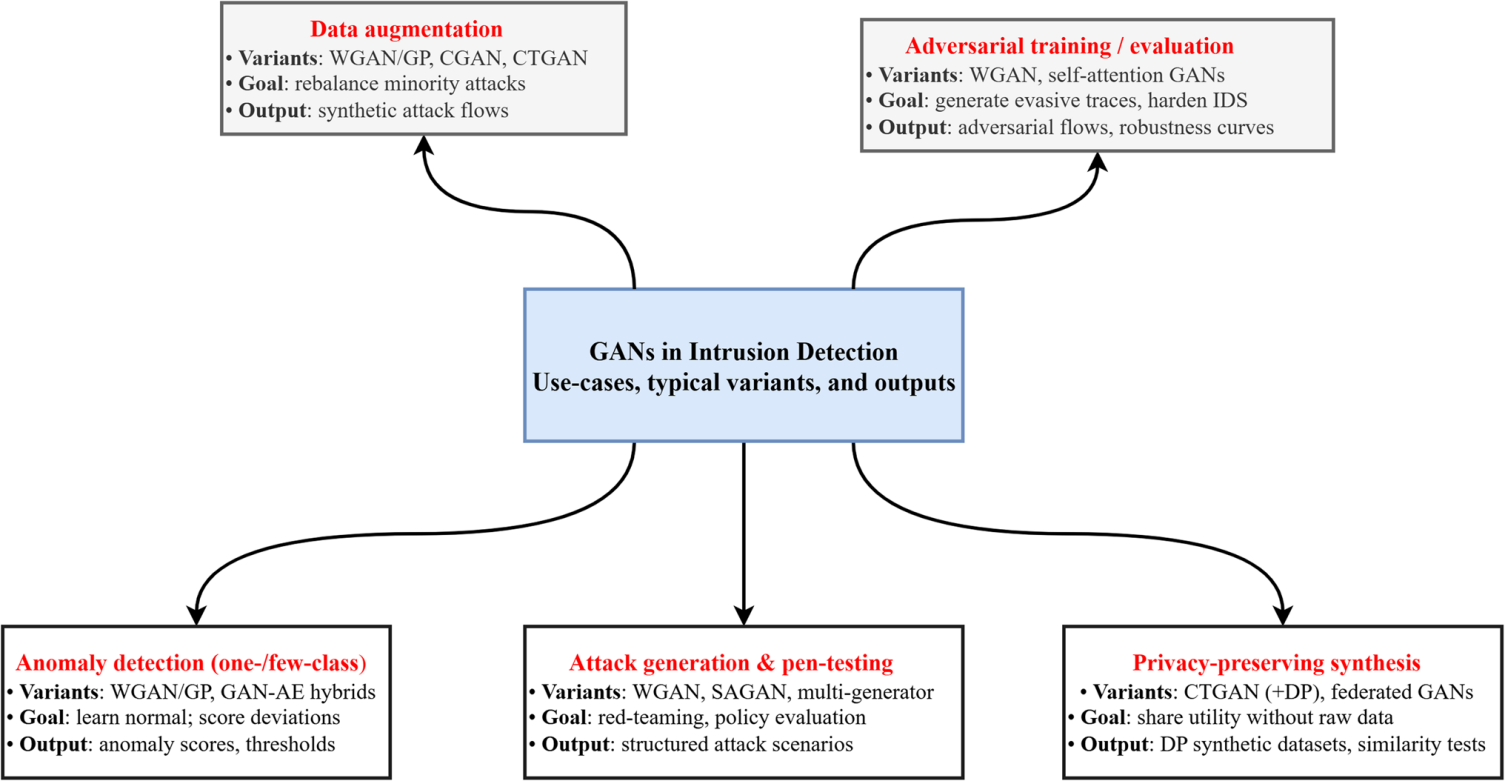
Taxonomy of GAN uses in IDS: the five main roles and their characteristic variants and outputs

To situate the rest of the survey, Fig. 2 maps the principal ways GANs are employed within IDS pipelines. We distinguish five use cases: data augmentation, adversarial training/evaluation, anomaly detection, privacy-preserving synthesis, and attack generation for penetration testing. Each use case aligns with characteristic GAN variants and produces different artifacts (e.g., synthetic flows, adversarial traces, or reconstruction scores), which we analyze in the following sections.

In addition to the taxonomy in Fig. 2, and 3 depicts a generic architecture of a GAN-enhanced IDS. The generator is trained on minority or benign traffic to produce synthetic samples, the discriminator distinguishes real from generated data, and the resulting augmented or reconstructed traffic is passed to a downstream classifier (e.g., CNN, LSTM or gradient-boosted tree) that performs the final intrusion decision. This block diagram is used as a reference when we describe specific architectures in Sects. 4.5 and 5.

**Fig. 3:**
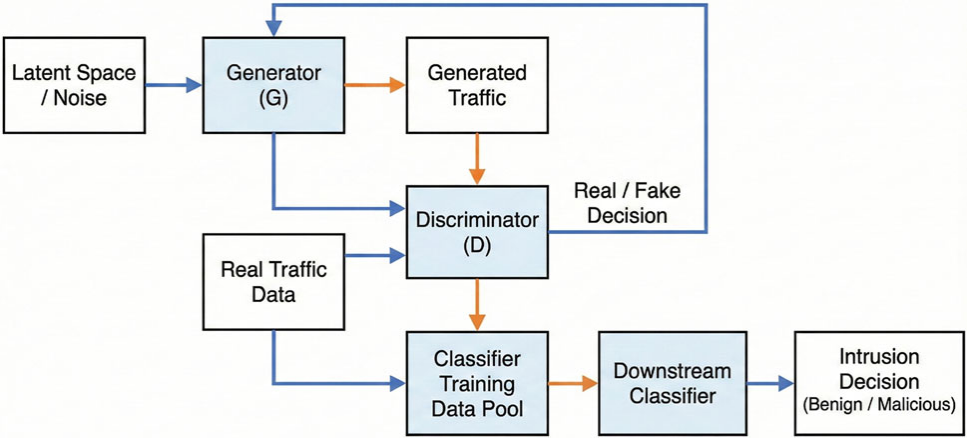
Generic architecture of a GAN-enhanced intrusion detection system. The generator produces synthetic or reconstructed traffic, the discriminator distinguishes real from generated samples, and a downstream classifier uses the resulting data to make intrusion decisions

Among the reviewed studies, many employed more than one GAN variant. Wasserstein GANs (WGANs) and Conditional GANs (CGANs) were the most commonly adopted variants. Deep Convolutional GANs (DCGANs) and self-attention GANs also appear in several studies, while Tabular GANs (TGAN/CTGAN) are used less frequently. Because individual studies sometimes combine multiple GAN variants, the categories in Table 1 are not mutually exclusive.

Deep Convolutional GANs (DCGANs) are commonly used for image-based representations of network traffic or malware. Self-attention mechanisms are adopted to better capture temporal dependencies in network flows. Tabular GANs (TGAN/CTGAN), specifically designed for tabular IDS data, appear mainly in recent work.

Table 1 summarizes the main GAN variants used in IDS research.

**Table 1:** Comparison of GAN architectures for intrusion detection

Architecture	Primary applications	Strengths	Limitations
WGAN [5, 12, 24]	Generating diverse attack samples; addressing mode collapse	Improved training stability and gradient flow, producing high-quality synthetic attack samples to rebalance datasets. Variants such as EO-WGAN and other multi-generator Wasserstein models further enhance minority-class detection and stability [24]	More computationally demanding due to the critic network and gradient penalty regularization; enhanced variants add additional computational cost
CGAN [13, 14]	Targeted generation of specific attack types; addressing class imbalance	Uses class labels to guide generation, thus helping synthesize rare attack classes and improve recall. Recent extensions (e.g., CE-GAN) integrate aggregation encoders to boost diversity and recall	Requires labeled data; performance may degrade on extremely rare classes; extended variants introduce extra complexity and tuning parameters
DCGAN [15, 25]	Image- or feature-based malware/intrusion representation	Learns hierarchical features via convolutional layers; can generate new samples to address class imbalance	Less suitable for sequential or temporal traffic and prone to mode collapse on complex patterns
SAGAN [7, 17]	Modeling complex temporal/sequential patterns	Attention mechanisms capture long-range dependencies, enabling realistic adversarial flows	Higher computational cost and model complexity
CTGAN [5, 9, 20]	Tabular network traffic and feature-based data	Designed for mixed discrete–continuous tabular data, aiding minority-class oversampling. Extensions such as CTGSM-DNN combine CTGAN with SMOTEENN to improve rare-class detection [26]	Fewer mature implementations; tuning can be challenging
ACGAN [18]	Multi-class attack generation; joint classification	Auxiliary classifier improves sample fidelity and allows simultaneous class prediction	More complex loss can cause per-class mode collapse if not tuned correctly
Hybrid/Specialized	Multi-generator or domain-specific variants (e.g., TMG-GAN [27], Recombination GAN [28], RGAN, EO-WGAN [24])	Combine GANs with oversampling, attention or multi-generator designs to improve diversity, stability and minority-class coverage	Custom architectures often require problem-specific tuning and may not generalize across datasets; increased architectural complexity can impact training time

### Wasserstein GANs for Intrusion Detection

Among the various GAN architectures, Wasserstein GANs (WGANs) [12] have gained significant traction in security research due to their training stability and improved quality of generated samples. WGANs replace the original GAN’s Jensen–Shannon divergence-based objective with the Earth Mover’s (Wasserstein) distance, which provides a more meaningful gradient even when the real and generated distributions have minimal overlap.

Araujo-Filho et al. [1] combine self-attention with temporal convolutions to capture short- and long-range dependencies (see Section V.C for detection results). Zhao et al. [5] systematically compared vanilla GAN, WGAN and CTGAN augmentation on CIC-IDS2017. WGAN augmentation yielded the most consistent improvements for the under represented botnet class, raising recall from 0.46 to 0.81 and F1 from 0.60 to 0.90. VanillaGAN achieved comparable gains, while CTGAN improvements were modest. Kumar and Sinha [4] applied WCGAN with gradient penalty for minority-class generation, achieving significant performance gains across multiple datasets (detailed in Sect. 5.1).

Overall, the reviewed studies suggest that WGANs are well suited to security settings because their Wasserstein objective stabilizes training and improves gradient flow, enabling the generation of diverse yet realistic attack samples. This stability helps mitigate mode collapse and supports more reliable augmentation of minority attack classes in GAN-based IDS pipelines.

On the system level, Park et al. [29] combine a Wasserstein-based distance generator with a more advanced AI-dependent network intrusion detection system (AI-NIDS) that solves the problem of class imbalance in a variety of benchmarks. The WGAN component improves the diversity and fidelity of minority-class traffic used for downstream training, and empirical results indicate better generalization to modified or variant attacks relative to non-adversarial augmentation. This reinforces the practical utility of Wasserstein training for IDS pipelines beyond isolated data generation modules.

### Conditional GANs and Variants

The appeal of conditional GANs in security research is especially obvious in the case of severe class imbalance. Most of the network traffic is benign, and the attacks represent only a small fraction. In this regard, the conditional GANs (CGANs) are useful as they can produce class-conditioned minority samples, without simply duplicating the existing samples.

Several studies have sought to improve the generation of specific attack types through targeted architectural and training modifications. Babu and Rao [14] proposed MCGAN (Modified Conditional GAN), adapting the generator to better capture intrusion-relevant feature patterns and employing the Nadam optimizer to stabilize and accelerate training. Evaluated on the NSL-KDD+ dataset, MCGAN produced notable gains for previously difficult minority classes, including Remote-to-Local (R2L) and User-to-Root (U2R), where detection rates had been reported below 40% prior to augmentation. Building on this direction, Rao and Babu [22] introduced an Imbalanced-GAN (IGAN) framework to generate synthetic minority attack samples; when coupled with CNN–LSTM models, the resulting system achieved over 98% accuracy (see Sect. 5.1 for details).

More recent work has also emphasized conditional generation for tabular network security data. Alabsi et al. [9] applied a Conditional Tabular GAN (CTGAN) to IoT intrusion detection, generating synthetic minority-class IoT attack instances and incorporating them into the training of a deep neural network IDS. In comparison with conventional oversampling techniques such as SMOTE, CTGAN-based augmentation maintained detection accuracy above 98% while yielding materially higher recall for rare attack categories. Similarly, Mouyart et al. [30] used CTGAN to expand insider-attack samples for a reinforcement learning–based, multi-agent IDS, reporting an insider threat recall of approximately 86%, exceeding baseline performance.

Collectively, these findings indicate that CGANs and their tabular variants can mitigate class imbalance effects in intrusion detection by enabling targeted synthesis of underrepresented attack types, thereby supporting more balanced training distributions and more uniform detection performance across classes.

### Self-Attention GANs and Temporal Models

Self-Attention GANs (SAGANs) [17] incorporate attention mechanisms to capture long-range dependencies in data, making them particularly suitable for modeling complex network traffic patterns with temporal characteristics. These architectures have shown promise for intrusion detection applications that involve sequential or time-series data.

Araujo-Filho et al. [1] combined self-attention with temporal convolutions to capture both short- and long-range dependencies, improving detection of complex temporal attack patterns (see Sect. 4.3 for performance results).

Aldhaheri and Alhuzali [7] developed SGAN-IDS, a framework that uses a self-attention GAN to generate adversarial network flows for testing IDS resilience. The self-attention component enabled the GAN to model sophisticated attack strategies that consider long-term packet sequences. Their experiments showed that SGAN-IDS reduced the detection rate of five state-of-the-art ML-based IDSs by an average of 15.93%, successfully crafting adversarial flows that evaded detection. Luo and Wan [28] proposed a Recombination Generative Adversarial Network (RGAN) for intrusion detection, which employed a DCGAN with self-attention in its first stage, alongside a GRU-based classifier. This approach enabled the model to capture both spatial and temporal features in network traffic, improving F1-scores for rare attacks by approximately 5–10% over baseline classifiers on the CSE-CIC-IDS2018 dataset.

The experiments highlight the practicality of the self-attention processes in generative adversarial network (GAN) systems in intrusion detection. Self-attention GANs (SAGANs) and their variants can be used to synthesize more realistic attack patterns and detect complicated attack patterns that can evade a simple model by learning long-range dependencies and temporal relationships in network traffic. This expertise is particularly decisive to advanced persistent threats (APTs) and complex attack campaigns with lengthy periods of service.

### Specialized GAN Architectures

As GAN-based intrusion detection matured, researchers extended standard architectures to meet domain-specific requirements—such as exploring quantum generative models, integrating evolutionary optimization, or modeling security-relevant data types (e.g., URLs). These specialized designs aim to address limitations of general-purpose GANs in security settings.

**Fig. 4:**
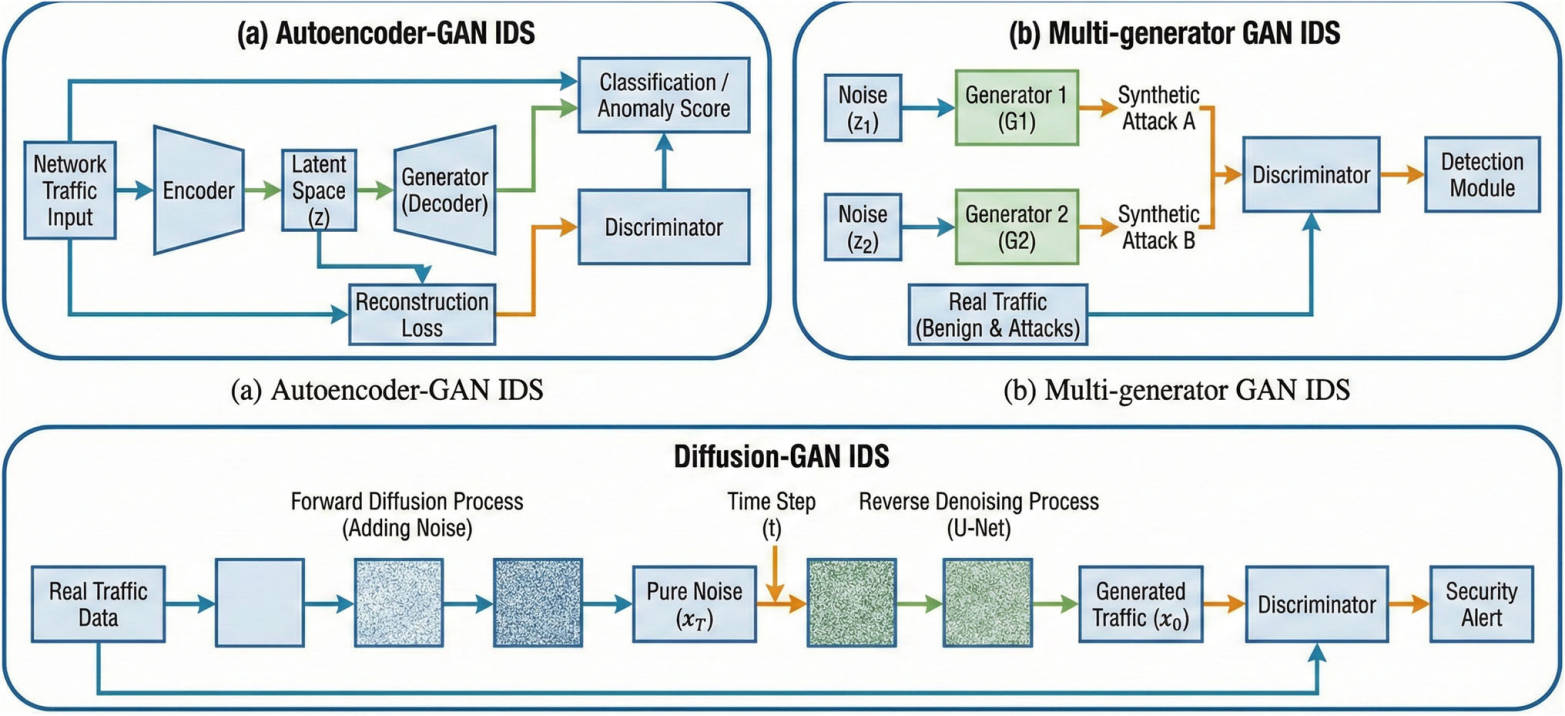
Representative specialized GAN-based IDS architectures, showing how additional modules such as autoencoders, multi-generator branches or diffusion sub-networks are integrated around the core generator–discriminator pair

Figure 4 summarizes a generic specialized GAN-based IDS architecture, showing how the generator and discriminator are combined with feature-extraction (e.g., autoencoder) and classification modules within the IDS pipeline.

Rahman et al. [23] explored Quantum Generative Adversarial Networks (qGANs) for intrusion detection, implementing a qGAN using IBM Qiskit to generate network traffic patterns. While largely conceptual and constrained by current quantum hardware limitations, their work suggests that quantum generators might craft more complex adversarial examples, potentially improving zero-day attack detection.

Rahman et al. [23] explored quantum GANs (qGANs) for intrusion detection using IBM Qiskit. While largely conceptual due to current hardware constraints, their work suggests that quantum generators may model complex distributions relevant to advanced threats. Singh et al. [16] combined DCGAN-based augmentation with a ResNet classifier optimized via Glowworm Swarm Optimization, reporting improved detection on NSL-KDD. Shuang et al. [6] proposed attackGAN, a Wasserstein GAN framework that crafts evasive traffic using IDS feedback and reports higher attack success than baseline GAN and gradient-based attacks (FGSM, PGD, CW). Rahman et al. [31] introduced SYN-GAN, training an IoT IDS solely on synthetic attack traffic; on ToN_IoT they report improved minority-class accuracy, although confidence intervals and statistical significance were not reported.

These specialized architectures reflect the diverse requirements of intrusion detection across different contexts and attack types. They also demonstrate the versatility of the GAN framework, which can be adapted and extended to address specific security challenges through innovative combinations with other techniques such as quantum computing, evolutionary algorithms, variational autoencoders, and transformer models.

#### Hybrid GAN–Autoencoder and Multi-Generator Models

A recent hybrid architecture couples a Wasserstein GAN with an autoencoder (WGAN-AE). By using the WGAN for stable training and the autoencoder to extract salient features, this model achieves PR–AUC up to 99.8% on the 5GNIDD dataset and 97.35% accuracy on the IDSIoT2024 dataset, with memory footprints around 60 kB [32]. Another approach, TMG-GAN, employs multiple generators and a classifier; each generator synthesizes a different attack class, while a cosine-similarity loss encourages diversity. TMG-GAN improves precision, recall and F1-scores on CIC-IDS2017 and UNSW-NB15 compared to single-generator GANs and traditional oversampling [27].

#### Diffusion and Quantum Generative Models

Recent work has expanded beyond GANs to consider alternative generative paradigms. Diff-IDS converts network features into grayscale images, augments them via flipping, and trains a Unet-based diffusion model; a feature-masking algorithm then enhances representation. Diff-IDS achieves high detection accuracy and training efficiency on CIC-IDS2017, NSL-KDD and KDD99 [33]. Diffusion models have also been used for adversarial purification: Merzouk et al. [34] show that diffusion-based purification can remove adversarial perturbations, identifying optimal diffusion noise and step parameters to maximize robustness. Beyond diffusion, quantum generative adversarial networks (QGANs) leverage variational quantum circuits; Hammami et al. [35] demonstrate a QGAN for multivariate time-series anomaly detection that attains high accuracy and F1-scores with only 80 parameters and remains effective under noise. These alternatives offer promising directions for resource-constrained or adversarial settings.

#### Recent Peer–Reviewed Results (2024–2025)

A series of fresh studies further confirm that *stability–oriented* GAN variants materially help IDS under class imbalance. Zhao et al. evaluate three generators—vanilla GAN, WGAN, and CTGAN—for augmenting CIC-IDS2017 and show that WGAN-based augmentation yields the most consistent lift for underrepresented botnet traffic. When the IDS was trained with $99\times$ WGAN-generated botnet samples and tested on the original class, precision/recall/F1 reached 1.00/0.81/0.90, improving recall by $35\%$ and F1 by $30\%$ over the baseline without augmentation [5].

Beyond architecture choice, conditional designs that explicitly preserve minority-class characteristics continue to advance. Yang et al. introduce CE-GAN, a Conditional GAN with an aggregation encoder–decoder and a composite loss to jointly preserve authenticity and diversity of synthetic flows; on NSL-KDD and UNSW-NB15 it significantly improves minority-class metrics while maintaining overall accuracy [36].

## GAN Applications in Intrusion Detection

The number of available studies varies substantially across application areas. Data augmentation and adversarial training have attracted many more contributions than, for example, SDN-based or diffusion model IDS. In the following subsections, we therefore (i) select representative papers for mature topics and (ii) include all peer-reviewed work we could identify for emerging topics, such as SDN, privacy-preserving GANs and diffusion/quantum models. This leads to an uneven number of citations per topic but accurately reflects the current state of the literature.

### Data Augmentation for Imbalanced Datasets

Class imbalance has plagued intrusion detection since the field began. In any real network, malicious traffic makes up a tiny fraction of overall activity. Some attack types are so rare that you might see only a handful of examples in months of data collection. This skew can bias machine learning models toward the majority class, reducing recall for minority attacks and increasing the risk of missed detections for rare but critical threats. Traditional approaches to this problem involve duplicating existing samples or using techniques like SMOTE to interpolate between known examples. But GANs offered something more appealing: the ability to generate entirely new attack instances that preserve the essential characteristics of each attack type while adding realistic variation.

**Fig. 5:**
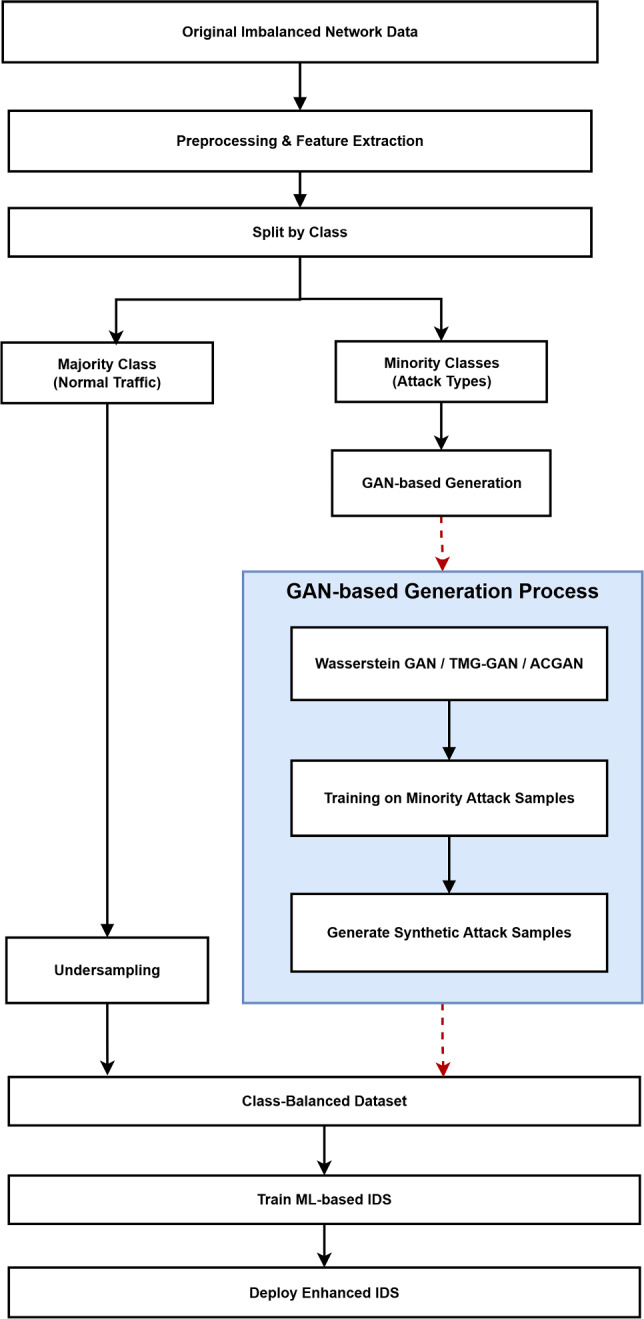
Overall pipeline for using GAN-based data augmentation in IDS: from raw traffic through class-balanced training data to the training and deployment of an enhanced intrusion detector

Figure 5 illustrates a typical data augmentation pipeline in GAN-based intrusion detection systems. First, the initial imbalanced data is pre-processed in order to normalize the representations of features and remove noise. Then, a GAN, or a collection of specialized GANs, is trained on classes of minority attacks, and allows the model to encode the underlying distribution of underrepresented events. The trained GAN produces synthetic attack samples on convergence and these samples are combined with the original sample to create a balanced training sample. Lastly, the intrusion detection classifier gets trained on this augmented dataset and thus the detection performance is improved especially to the attack type that has been underrepresented.

The multi-generator approach that Ding’s team [27] developed tackles a fundamental question: should you use one GAN to generate all attack types, or specialized GANs for each? Their TMG-GAN (also referred to as TMG-IDS) architecture assigns a dedicated generator to each minority attack family while using a shared discriminator that also handles classification. The reasoning makes sense: different attack types have fundamentally different characteristics, so why force a single generator to learn everything? On CIC-IDS2017 and UNSW-NB15, the system reports high overall precision/recall/F1 and consistent macro-F1 gains over single-generator baselines, indicating better minority-class coverage without sacrificing performance on majority traffic. This result supports the view that Wasserstein training, when paired with class-specialized generators, can materially mitigate mode collapse and skew in IDS augmentation settings.

Kumar and Sinha [4] demonstrated the effectiveness of a Wasserstein Conditional GAN (WCGAN) in generating minority-class attacks to improve detection. Their experiments on NSL-KDD, UNSW-NB15, and BoT-IoT datasets showed that augmenting training data with GAN-synthesized attacks significantly boosted precision, recall, and F1-score. The proposed WCGAN-XGBoost approach outperformed both vanilla oversampling and prior conditional GAN methods, achieving over 95% detection accuracy across attack classes.

Rao and Babu [22] addressed skewed class distributions with an Imbalanced-GAN (IGAN) framework that generates synthetic minority attack instances (e.g., infiltration, U2R attacks) to balance training sets. Their approach, combining the IGAN with a hybrid LeNet-5 CNN and LSTM network, achieved $>98$% accuracy on UNSW-NB15 and CIC-IDS2017, markedly improving recall for rare attack categories. The authors highlight that an even mix of real and GAN-generated data yielded the best classifier performance, matching an equivalent fully real dataset.

Mouyart et al. [30] used a Conditional Tabular GAN (CTGAN) to generate additional insider attack samples and address dataset imbalance in a multi-agent IDS. Testing on the CERT 4.2 insider threat dataset, the GAN-augmented RL IDS achieved a high insider threat recall of  86%, outperforming a baseline RL IDS that suffered on sparse attack classes.

The experimental results presented by Zhao et al. [5] provide an interesting fact about the generation of synthetic data: Small oversampling ratios to the WGAN framework, such as the multiplicative factor, are approximately $4\times$, give rise to most of the performance gains, with oversampling factors beyond giving diminishing returns. Although CTGAN shows some advantages when expanding to larger oversampling ratios, CTGAN is consistently underperforming against WGAN in the same experiment setting. These results have important implications for computational efficiency because this shows that data practitioners need not synthesize large quantities of synthetic data to see meaningful improvements.

For IoT settings with extremely rare attacks, Menssouri and Amhoud propose a two-stage pipeline (CTGSM-DNN): a CTGAN first synthesizes minority classes, then SMOTEENN refines the augmented set. On CSE-CIC-IDS2018, they obtain $99.90\%$ overall accuracy and *80%* accuracy on rare classes—evidence that conditional tabular generators combined with classical rebalancing can materially reduce miss rates for the scarcest attacks [26]. In [9], Alabsi et al. employ a Conditional Tabular GAN to synthesize DoS/DDoS traces in BoT-IoT. CTGAN’s conditioning and tabular design handle mixed discrete and continuous features while preserving feature dependencies relevant to intrusion semantics. Compared with non-augmented training (and classical resampling), the CTGAN-augmented pipelines achieve higher accuracy/recall/F1 on DoS/DDoS, with limited false-positive inflation, underscoring CTGAN’s suitability for feature-rich IoT telemetry where rarity and heterogeneity of attacks are pronounced.

Beyond standard CGANs, recent ACGAN-based designs aim to preserve minority semantics during rebalancing. SPE-ACGAN introduces sample-preserving enhancements to the auxiliary classifier GAN, generating class-conditional flows that improve recall and F1 for underrepresented attacks while maintaining overall accuracy on CIC-IDS2017 and UNSW-NB15 [19]. The auxiliary classification objective encourages the discriminator to retain discriminative features for rare classes, yielding more faithful minority samples than naive oversampling.

Recent research by Yang [37] applied a GAN-based data resampling approach to address class imbalance in wireless sensor network intrusion detection. The method couples GAN-generated synthetic samples with an improved spatiotemporal residual network that employs multi-scale one-dimensional convolutions, gated recurrent units and identity mapping. Experiments on the NSL-KDD, UNSW-NB15 and CIC-IDS2017 datasets achieved accuracies of 99.62%, 83.98% and 99.86%, respectively, demonstrating that GAN-assisted resampling combined with spatiotemporal feature learning can yield competitive performance.

Most studies reviewed report that GAN-based augmentation improves classification metrics for minority attack classes compared with simple oversampling methods such as SMOTE. This benefit arises because GANs can approximate the distribution of minority classes rather than duplicating existing samples [38]. Nevertheless, some authors note diminishing returns when augmentation ratios are very high or when the discriminator overfits the synthetic data; therefore, the effectiveness of GAN augmentation depends on careful tuning.

### GANs for Adversarial Training of IDS

GANs have also been used to support *adversarial training* for intrusion detection, where the goal is to expose a detector to inputs that are intentionally hard to classify. Instead of training only on collected attack traces, a generator is trained to produce traffic variants that increase the error rate of a target IDS. These adversarial samples can then be used to diagnose weaknesses and, in some studies, to retrain the detector to improve robustness under the assumed threat model.

Wang et al. [8] illustrated this idea with IDS-GAN. In their setup, the generator learns small perturbations that convert benign traffic into adversarial examples while aiming to keep the resulting traffic plausible. They report that conventional IDS models can be evaded at high rates (over 95% in their experiments) without obvious loss of traffic realism, suggesting that accuracy on standard test splits may overstate practical robustness.

Shuang et al. [6] proposed attackGAN, using a Wasserstein-based objective to improve training stability and the quality of generated adversarial samples. Their method uses feedback from the target IDS to guide generation, allowing the adversary to adapt to the detector during training. On NSL-KDD, they evaluated five IDS architectures and reported higher attack success than several baselines. For example, against an SVM-based IDS, attackGAN achieves an attack success rate of 81.3% (vs. 40.87% for a baseline GAN in the same setting). Under their Naive Bayes evaluation, they also report higher success than FGSM (17.76%), PGD (29.78%), and CW (21.25%).

Data scarcity is another practical constraint, especially for rare attacks and emerging behaviors. Randhawa et al. [39] introduced an Evasion Generative Adversarial Network intended to operate in low-data regimes. The central motivation is to maintain sample realism and diversity when only limited training examples are available, and they report improvements over standard GAN training under small-dataset conditions.

The same adversarial training logic has been applied outside network traffic. Kamran et al. [40] studied phishing URL detection using a semi-supervised conditional GAN in a game-theoretic formulation. The generator attempts to produce convincing phishing URLs while the discriminator (and downstream detector) learns to separate malicious from legitimate patterns. In their experiments, the resulting detector achieves approximately 98% accuracy against simulated zero-day phishing attempts.

More recent work has focused on increasing the variety of adversarial samples, since limited diversity can reduce the practical value of adversarial training. Xu et al. [41] proposed DEMGAN, combining multiple generators with distortion enhancement mechanisms to mitigate mode collapse. They report evasion rates of 97.42% and 87.51% across different evaluation datasets, and they further report that retraining with these adversarial examples increases detection rates by 86.78% (under their evaluation protocol).

Overall, GAN-based adversarial generation highlights a dual-use tension: the same tools that produce effective evasive traffic can also be used to stress-test IDS models and to support adversarial retraining. In practice, the defensive value depends on how realistic the generated samples are, how the threat model is defined, and whether robustness gains transfer to unseen attacks and deployment conditions.

### GANs as Anomaly Detectors

In addition to supporting augmentation or adversarial training, GANs can be used *as* anomaly detectors for intrusion detection. A common design trains the GAN primarily on benign traffic so it learns a model of normal behavior. At inference time, deviations from this learned distribution are treated as potential intrusions, using either the discriminator score, a reconstruction-based discrepancy, or related anomaly scores.

Araujo-Filho et al. [1] presented an unsupervised GAN-based IDS that combines Temporal Convolutional Networks with self-attention. The generator is trained to model benign network traffic, and the discriminator is used to flag anomalous samples as intrusions. They report improved accuracy and substantially lower latency, with the GAN-IDS running $3.8\times$ faster than state-of-the-art LSTM-based IDSs in their comparison, which is relevant for settings where detection delay is a primary constraint.

Luo and Wan [28] proposed a Recombination GAN (RGAN) that uses adversarial training in a two-stage pipeline alongside a classifier. Their method first trains a self-attention DCGAN to generate attack traffic and a GRU-based classifier, then refines the generator and classifier jointly. On CSE-CIC-IDS2018, they report F1-score gains of roughly 5–10% for rare attacks compared with baseline classifiers. While the pipeline is not purely one-class anomaly detection, it reflects a related goal: improving recognition of low-frequency behaviors without substantially increasing false alarms.

Kim and Pak [42] explored a one-class, GAN-assisted gating mechanism that operates on packet-level features to support *early* decisions, before full sessions are available. The GAN component checks whether early packets match learned normal patterns, enabling faster alerts while maintaining performance that the authors report as competitive with session-level baselines. This emphasis on time to alarm is particularly relevant for edge deployments and inline enforcement.

Iliyasu and Deng [43] proposed N-GAN, a weakly supervised anomaly detector designed for situations where only a small fraction of malicious samples is labeled. Evaluated on CIC-IDS2017, they report that N-GAN adapts to evolving normal patterns and achieves higher detection rates than reconstruction-based anomaly detectors while keeping false positives lower.

The practical advantage of applying GAN-based anomaly detection is that it is capable of detecting classes of behavior not directly indicated by the training data, potentially useful in identifying new or zero-day attacks. Nevertheless, such systems typically necessitate the detection thresholds and continuous adaptation. In case of changes in normal traffic patterns (a concept referred to as concept drift), the system will generate a higher number of false positives unless the model’s definition of normal behavior is updated periodically on a consistent basis.

### GANs for Attack Generation and Penetration Testing

GANs can be used to generate highly realistic malicious traffic for controlled testing of intrusion detection systems. Such synthetic traces can help identify detector weaknesses and support defensive hardening before similar behaviors appear in operational environments.

One illustration is SGAN-IDS, proposed by Aldhaheri and Alhuzali [7], which uses a self-attention GAN to craft adversarial network flows intended to probe IDS resilience. The generator is trained to reproduce malicious patterns aligned with evolving attack behavior. In evaluation, the synthetic flows reduced detection rates across five state-of-the-art machine learning IDSs by an average of 15.93%, indicating that GAN-driven simulation can reveal concrete robustness gaps.

Mari et al. [3] employed a deep GAN that was trained on the NSL-KDD data to create adversarial attack traffic. The produced samples that contained realistic DoS and probe events were able to bypass the detector in most of these cases, and certain types of attacks were able to go undetected virtually. With the addition of these samples to the training set, the detection of historically poor classes improved, particularly, R2L and U2R.

Empirical research in domains indicates that the traditional testing can fail to detect the vulnerabilities that arise during adversarial generation. Mbow et al. [44] worked with the environments of the IoT and trained a GAN repeatedly to produce malicious IoT packets that were detected by an anomaly detector as benign. The resultant traces led to severe performance impairment and the accuracy decreases of up to 20% on some types of attacks. Their results highlight the necessity to test IoT IDSs on adversarial inputs.

Adversarial generation has also been extended beyond network flows to complex software artifacts. Doan et al. [45] introduced AAGAN, a GAN-based framework for generating Android malware variants (adversarial APKs) that target malware classifiers. They reported that generated samples changed the predictions of state-of-the-art detectors in 99% of cases while preserving application functionality and maintaining close visual similarity to the original apps. Although repeated adversarial retraining reduced evasion success, the attack remained highly effective (approximately 89% after five rounds), suggesting persistent difficulty in achieving robust defenses in this context.

Taken together, these studies sharpen the dual-use considerations noted in Section I: generative methods that enable rigorous, proactive evaluation can also be repurposed for misuse. This trade-off supports the case for careful experimental governance, responsible disclosure, and defenses explicitly designed to handle adversarially generated inputs.

### GAN-Based IDS in SDN/Programmable Networks

Recent work embeds GAN-based detection inside multilayer SDN defenses. Nayak and Bhattacharyya combine Four-Q curve (elliptic-curve) MAC authentication, univariate ensemble feature selection, and a Dual Discriminator Conditional GAN (DDcGAN) to classify SDN traffic into normal, assault, and suspect flows. On their testbed, the system achieves $98.29\%$ accuracy (F1 0.975; precision $95.8\%$) with a true-positive rate of $99.04\%$ at $50\%$ malicious nodes and a false alarm rate of $2.05\%$, while also reducing power consumption by $4.5\%$ relative to baselines [46]. These results indicate that adversarially trained generators can be engineered to meet performance and efficiency requirements in programmable networks.

### Privacy-Preserving GANs for Security Data

Privacy concerns often limit the sharing and use of network traffic data for intrusion detection research and development. GANs offer a potential solution by generating synthetic data that preserves the statistical properties of real data without exposing sensitive information.

Privacy utility with formal controls. A 2024 study by Alabdulwahab et al. [21] integrates differential privacy into a CTGAN for IoT sensor IDS data and adds distributional controls (dynamic adjustment and quantile matching). Following the study’s convention of reporting the KS complement ($1{-}D$; higher indicates greater similarity), they obtain a KS score of 0.80, showing the synthetic traffic closely matches the real distribution. Importantly, IDS detection performance remains near the non-private baseline, indicating a favorable privacy–utility trade-off while mitigating singling out, linkability, and inference risks.

Aceto et al. [47] propose a conditional variational autoencoder (CVAE) to synthesize anonymized network traffic traces for NIDS training. They show that synthetic data can be used to train a classifier with limited F1-score loss: when training on synthetic data, the F1-score drops by 12.35% for the IoT-23 dataset, 0.51% for KITSUNE and 3.83% for IDS2018. The Jensen–Shannon divergence between synthetic and real distributions is about 0.1 and the F1-score loss for the IDS2018 dataset is at most  1.25%, indicating that the synthetic traces closely match real traffic distributions.

Jiang et al. [48] proposed VertiGAN, a distributed GAN-based privacy-preserving publication method for vertically partitioned data. Their protocol allows multiple parties to jointly train a GAN on vertically partitioned data (each party has different attributes for the same individuals). Differential privacy mechanisms are integrated to protect each party’s data during GAN training. VertiGAN produced a synthetic fused dataset that preserved correlations across parties’ features, allowing effective anomaly detection on the combined data. It satisfied strong DP guarantees ($\epsilon < 1$) for each party’s input.

Hassan et al. [49] introduced HE-GAN, a differentially private GAN using Hamiltonian Monte Carlo based exponential mechanism. Rather than directly injecting noise in the training gradients, HE-GAN uses the exponential mechanism on a posterior derived from a private classifier and employs Hamiltonian Monte Carlo (HMC) to sample latent vectors for the GAN generator. By avoiding noise in the discriminator’s updates, HE-GAN mitigates the model degradation common in DP-GANs. Experiments on MNIST and Fashion-MNIST showed HE-GAN maintained downstream classification accuracy equivalent to non-private GAN training, and often better than traditional DP-GAN methods.

These approaches demonstrate the potential of privacy-preserving GANs to enable collaborative security research and development without compromising sensitive network data. By generating synthetic datasets that maintain the essential characteristics for intrusion detection while protecting privacy, these methods can facilitate knowledge sharing and cooperative defense strategies across organizations and sectors.

## Evaluation and Performance Analysis

### Datasets for GAN-Based Intrusion Detection

The evaluation of GAN-based intrusion detection systems relies heavily on benchmark datasets that capture diverse network behaviors and attack patterns. Our survey indicates that a handful of public datasets dominate in recent GAN-IDS research. Table 2 summarizes these datasets, the attack categories they encompass, and how they have typically been employed in GAN-based studies.

**Fig. 6:**
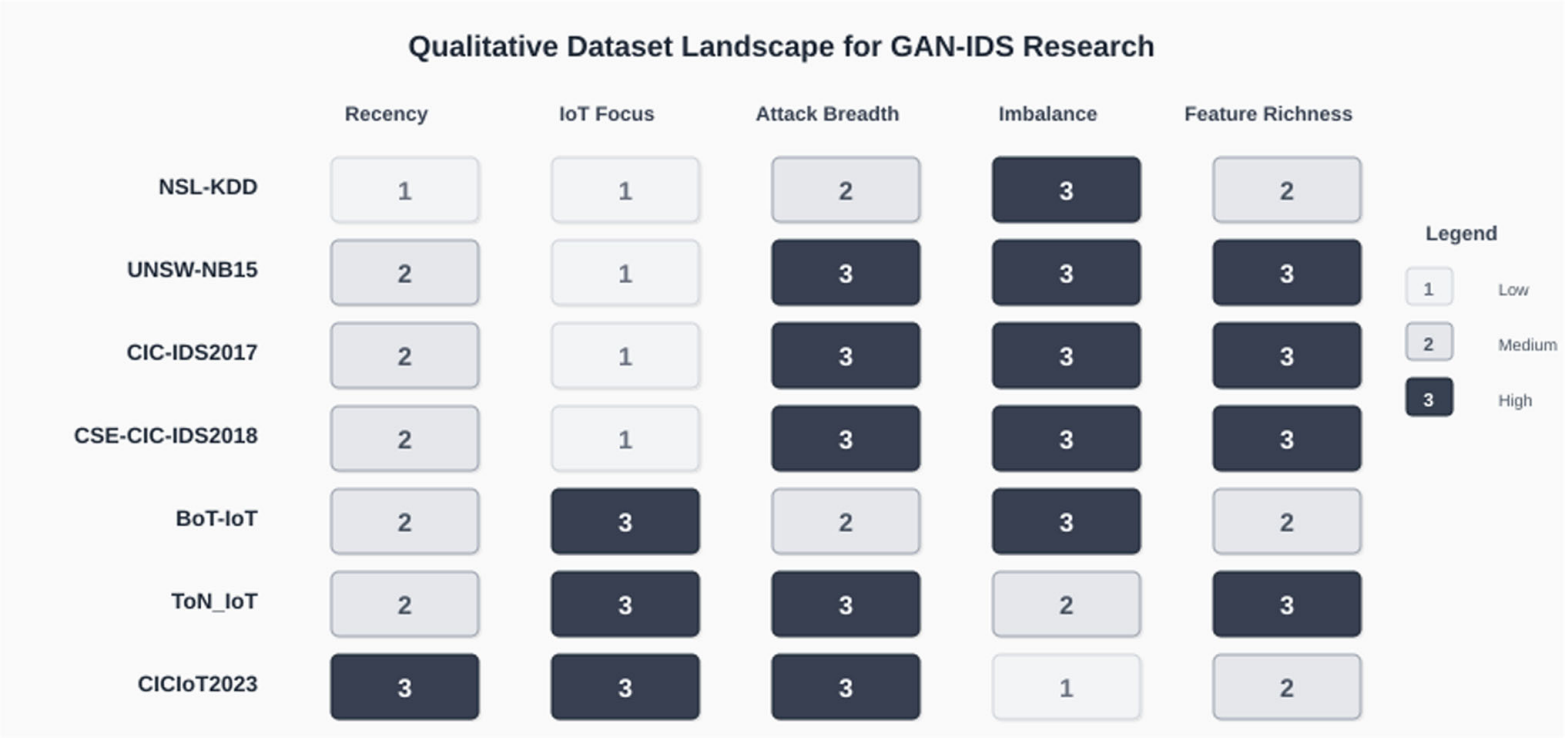
Qualitative dataset landscape for GAN-IDS research across five axes: recency, IoT focus, attack breadth, imbalance severity and feature richness. Values are encoded as 1 (low), 2 (medium) and 3 (high)

Figure 6 qualitatively contrasts widely used datasets along five axes relevant to GAN-IDS research—recency, IoT focus, attack breadth, imbalance severity, and feature richness. Each cell now contains an integer from 1 to 3 (1 = low, 2 = medium, 3 = high) to indicate the qualitative level along that axis; darker shading is retained only as a secondary visual cue for readers viewing the figure in color.

**Table 2:** Commonly used datasets in GAN-based intrusion detection research

Dataset	Attack categories (as reported)	Representative GAN usage in IDS research
NSL-KDD	39 attack types grouped into DoS, Probe, R2L and U2R [50]	Conditional and Wasserstein GANs synthesize rare R2L/U2R to balance training and boost minority recall. Improved spatiotemporal ResNet with GAN resampling raises accuracy to 99.62% on NSL-KDD [37]
UNSW-NB15	Modern traffic with nine categories including Fuzzers, Analysis, Backdoor, DoS, Exploits, Generic, Reconnaissance, Shellcode and Worms [51]	IGAN/WCGAN often generate minority attacks (e.g., Shellcode, Worms) to improve recall and overall accuracy. Enhanced WGAN oversampling (EO-WGAN) achieves up to 95.2% accuracy with balanced precision and recall [24]; variants such as NF-UNSW-NB15 are used to evaluate adaptive resampling approaches
CIC-IDS2017	Benign + multiple attack scenarios: brute force, DoS, Heartbleed, web attacks, infiltration, botnet, DDoS [52]	GANs (vanilla, WGAN, CTGAN) augment underrepresented attacks; AttackGAN crafts adversarial flows for robustness studies; WGAN-augmented training improves botnet (precision = 1.00, recall = 0.81, and F1 = 0.90) [5]
CSE-CIC-IDS2018	Seven scenarios: brute force, Heartbleed, botnet, DoS, DDoS, web attacks, internal infiltration [52]	CTGAN and hybrids synthesize rare attacks (e.g., XSS/SQLi) and are combined with classical rebalancing (SMOTEENN); CTGSM-DNN yields 99.90% overall accuracy and $\sim$80% accuracy on rare attacks [26]
BoT-IoT	IoT traffic with DDoS/DoS, reconnaissance, keylogging and data exfiltration [53]	Conditional/Wasserstein GANs address skew; used to generate IoT botnet flows and adversarial traces for hardening
ToN_IoT	Normal + nine attacks: password cracking, scanning, ransomware, backdoor, DoS/DDoS, MITM, injection, XSS [54]	Comprehensive multi-modal IoT/IIoT dataset; enables evaluation of AI-based security systems across heterogeneous data sources (telemetry, OS logs, network traffic)
CICIoT2023	Large-scale IoT dataset collected from a 105-device topology with 33 executed attack scenarios grouped into seven categories (e.g., DDoS/DoS, Recon, Web-based, brute force, spoofing, Mirai), alongside benign traffic [55]	Used for training and evaluating IoT-focused IDS (including GAN-based augmentation and federated IDS studies) under modern IoT traffic conditions

Additionally, CICIoT2023 was introduced as a large-scale IoT benchmark collected from a realistic IoT testbed and is widely used for evaluating IoT-focused intrusion detection pipelines [55]. Several studies have employed multiple datasets to evaluate their approaches. For instance, Kumar and Sinha [4] tested their WCGAN-XGBoost approach on NSL-KDD, UNSW-NB15, and BoT-IoT, demonstrating consistent performance improvements across different network contexts. Similarly, Rao and Babu [22] evaluated their IGAN framework on both UNSW-NB15 and CIC-IDS2017, showing robustness across diverse traffic patterns.

A notable trend in recent research (2023–2024) is the increasing use of IoT-specific datasets to address the unique security challenges in IoT environments. Studies by Alabsi et al. [9], Rahman et al. [31], and Mbow et al. [44] specifically target IoT security using datasets like BoT-IoT and TON_IoT, reflecting the growing importance of this domain.

Despite the variety of available datasets, important limitations remain. NSL-KDD is still widely used due to its established attack taxonomy and broad adoption, but it has been criticized for not reflecting contemporary traffic patterns. Most datasets exhibit severe class imbalance, which motivates GAN-based augmentation but also complicates evaluation. Moreover, static snapshots quickly become outdated as attackers evolve, limiting their usefulness for assessing zero-day detection.

### Evaluation Metrics

Table 3 summarizes the metrics used in GAN-based IDS studies. A diverse range of metrics is employed to evaluate GAN-based intrusion detection systems, reflecting the multifaceted nature of IDS performance assessment. Traditional classification metrics such as accuracy, precision, recall, F1-score and ROC-AUC are widely used to gauge overall performance. However, because intrusion datasets are often highly imbalanced, many studies emphasize per-class measures—particularly recall for minority attack classes—to ensure that GAN-generated samples improve detection where it is most needed.

**Table 3:** Evaluation metrics used in GAN-based intrusion detection research

Metric category	Specific metrics	Notes on usage in GAN-IDS research
Classification performance	Accuracy, precision, recall, macro and weighted F1-score, ROC-AUC	These metrics assess overall and per-class detection performance. Because malicious traffic is often a small fraction of total traffic, recall on minority attack classes and macro-F1-scores (averaging F1 across classes) are frequently highlighted to ensure that GAN augmentation improves detection of rare attacks
Attack-specific detection	Attack detection rate, false alarm rate, per-attack detection accuracy	Provides a finer-grained view of detector effectiveness across individual attack types. Particularly useful for evaluating whether GAN-generated samples enhance recognition of underrepresented attacks without inflating false positives
GAN quality assessment	Frechet Inception Distance (FID), Inception Score, Jensen–Shannon divergence, Wasserstein distance	Used to gauge the realism and diversity of synthetic data. These measures originate from image domains; adapting or developing network traffic-specific metrics remains an open research need
Computational efficiency	Training time, inference time, resource footprint	Relevant for real-time or resource-constrained deployments. Some studies compare the computational cost of GAN augmentation versus traditional oversampling or assess whether GAN-based detection models can meet latency requirements
Adversarial robustness	Evasion success rate, robustness to perturbations, recovery after retraining	Measures how well an IDS withstands adversarial traffic generated by GANs and how effective adversarial training or retraining is at restoring detection performance
Privacy metrics	Differential privacy bounds, membership/attribute inference success	Used in privacy-preserving GAN frameworks to quantify the risk of leaking sensitive information. Metrics help assess the privacy–utility trade-off when sharing or training on synthetic security data

Beyond accuracy and F1-score, these metric categories capture the broader goals of evaluating GAN-based intrusion detection systems (IDS). Attack-specific metrics indicate whether data augmentation improves detection of minority classes; GAN quality metrics gauge how realistic the generated traffic is; and computational, robustness, and privacy metrics help ensure that gains in detection performance do not come at the cost of efficiency, security, or sensitive data exposure.

Kumar and Sinha [4] identified improved F1-scores for rare attack classes (U2R, R2L) as a central benefit of their WCGAN approach. Likewise, Rao and Babu [22] reported higher recall for infiltration attacks on the CIC-IDS2017 dataset using their IGAN framework.

For evaluating the quality of GAN-generated samples, researchers employ metrics such as Frechet Inception Distance (FID), Inception Score, and statistical divergence measures (Jensen-Shannon, Wasserstein) between real and synthetic data distributions. Zhao et al. [5] used statistical similarity tests to assess how closely their GAN-generated attack traffic resembled real attacks. Alabdulwahab et al. [21] employed the Kolmogorov-Smirnov test to measure the similarity between real and synthetic IoT traffic data.

Adversarial robustness metrics are increasingly important, particularly in studies focusing on evasion attacks or adversarial training. Aldhaheri and Alhuzali [7] measured the reduction in detection rate across multiple IDS when exposed to SGAN-IDS-generated adversarial flows. Wang et al. [8] quantified IDS evasion success rates for their IDS-GAN approach.

Privacy metrics have emerged in recent studies on privacy-preserving GANs. Alabdulwahab et al. [21] evaluated their DP-CTGAN approach using membership inference attack success rates and differential privacy guarantees. Jiang et al. [48] assessed VertiGAN using formal differential privacy bounds ($\varepsilon$) and empirical privacy leakage measures.

Overall, the diversity of evaluation measures reflects the multiple objectives of GAN-based IDS research: improving detection performance (especially for minority classes), generating high-quality synthetic traffic, enhancing robustness, maintaining computational efficiency, and preserving privacy. Achieving these goals simultaneously remains challenging because improvements along one dimension can introduce trade-offs in others.

### Performance Benchmarks and Comparisons

Our analysis of the literature reveals several performance benchmarks for GAN-based intrusion detection systems across different application contexts.

For data augmentation, the WCGAN-XGBoost method (Sect. 5.1) achieved over 95% detection accuracy, with 15–20% improvement for U2R and R2L attacks.

Rao and Babu [22] reported that their IGAN framework, when combined with a hybrid LeNet-5 CNN and LSTM network, achieved over 98% accuracy on the UNSW-NB15 and CIC-IDS2017 datasets. Their study found that an even mix of real and GAN-generated data yielded optimal classifier performance, comparable to a dataset with equivalent real samples.

For GAN-based anomaly detection, Araujo-Filho et al. [1] demonstrated that their unsupervised GAN-IDS with Temporal Convolutional Networks and self-attention achieved higher accuracy and was $3.8\times$ faster than state-of-the-art LSTM-based IDS. This performance gain highlights the efficiency advantages of their architecture for real-time intrusion detection.

In the context of adversarial evasion and training, Aldhaheri and Alhuzali [7] showed that their SGAN-IDS reduced the detection rate of five state-of-the-art ML IDSs by an average of 15.93%.

Shuang et al. [6] evaluated attackGAN on the NSL-KDD dataset, preserving functionality. Their Wasserstein GAN model achieved higher attack success and evade increase rates than GAN-based, FGSM, PGD and CW attacks; for example, its attack success rate reached 81.37% versus 40.87% for a baseline GAN.

Table 4 distills representative findings from recent GAN-based intrusion detection studies. Rather than listing exact accuracy values, the table highlights qualitative improvements, better minority-class detection, reduced latency, enhanced adversarial robustness or effective synthetic data generation, and notes the datasets used.

**Table 4:** Representative GAN-based IDS studies and main performance trends

Application area	Study	Main observation	Datasets
Data augmentation	Kumar and Sinha [4]	WCGAN–XGBoost generates minority-class attacks and improves overall accuracy and recall vs. SMOTE and earlier GAN-based oversampling	NSL-KDD, UNSW-NB15, BoT-IoT
Data augmentation	Rao and Babu [22]	Imbalanced-GAN (IGAN) with a CNN–LSTM backbone increases recall on rare attacks when real and synthetic samples are evenly mixed	UNSW-NB15, CIC-IDS2017
Data augmentation	Zhao et al. [5]	WGAN-generated botnet samples improve CIC-IDS2017 botnet precision/recall/F1 from 0.87/0.46/0.60 to 1.00/0.81/0.90 with moderate oversampling ratios	CIC-IDS2017
Data augmentation	Yang [37]	GAN-assisted resampling plus an improved spatiotemporal ResNet boosts overall accuracy and F1 on three benchmarks, especially for minority classes	NSL-KDD, UNSW-NB15, CIC-IDS2017
Data augmentation (IoT)	Menssouri and Amhoud [26]	CTGSM-DNN (CTGAN+SMOTEENN) reaches 99.90% overall accuracy and $\approx$80% accuracy on rare IoT attacks, outperforming DNN+SMOTE	CSE-CIC-IDS2018
Anomaly detection	Araujo-Filho et al. [1]	Unsupervised GAN-IDS with temporal convolutions and self-attention detects anomalies more accurately and $\approx$3.8$\times$ faster than an LSTM-based IDS	CSE-CIC-IDS2018 (benign traffic)
Anomaly detection	Luo and Wan [28]	Recombination GAN with self-attention and a GRU classifier improves F1 on rare attacks by 5–10% compared with baseline detectors	CSE-CIC-IDS2018
Adversarial training	Aldhaheri and Alhuzali [7]	Self-attention GAN generates adversarial flows that lower the detection rate of several ML-based IDSs by $\approx$16%; retraining with these flows improves robustness	Multiple IDS models
Adversarial training	Shuang et al. (attackGAN) [6]	attackGAN (Wasserstein GAN) crafts black-box evasive traffic that outperforms FGSM/PGD/CW and a baseline GAN on NSL-KDD	NSL-KDD
Adversarial training	Wang et al. [8]	IDS-GAN generates adversarial network traffic achieving >95% evasion success; using these samples for retraining improves IDS resilience	CIC-IDS2017 and others
IoT / SDN	Rahman et al. (SYN-GAN) [31]	GAN-only synthetic attacks train an IoT IDS that performs competitively, alleviating scarcity of minority IoT attacks	TON_IoT and related IoT datasets
IoT / SDN	Nayak and Bhattacharyya [46]	Multilayer SDN security with MAC authentication and a dual-discriminator cGAN attains 98.29% accuracy, F1$\approx$0.975 and 2.05% false alarm rate, with reduced power consumption	SDN testbed
IoT / IIoT	Riaz et al. [24]	EO-WGAN (SMOTE+WGAN) improves anomaly detection accuracy to 95.2% and outperforms SMOTE and standalone WGAN at high imbalance ratios	UNSW-NB15 and IIoT benchmarks
Privacy preserving	Alabdulwahab et al. [21]	Differentially private CTGAN generates IoT traffic that preserves IDS performance at about 95% of the non-private baseline while providing privacy guarantees	IoT sensor network data

These benchmarks demonstrate the significant performance improvements that GANs can bring to intrusion detection systems. However, direct comparisons across studies are complicated by differences in datasets, evaluation metrics, and implementation details. The lack of standardized evaluation frameworks remains a challenge for the field, as noted by several researchers [10, 11].

## Challenges and Future Directions

### Technical Challenges in GAN-Based Intrusion Detection

Despite encouraging results, GAN-based intrusion detection systems still face technical challenges that limit practical deployment and operational effectiveness.

**Fig. 7:**
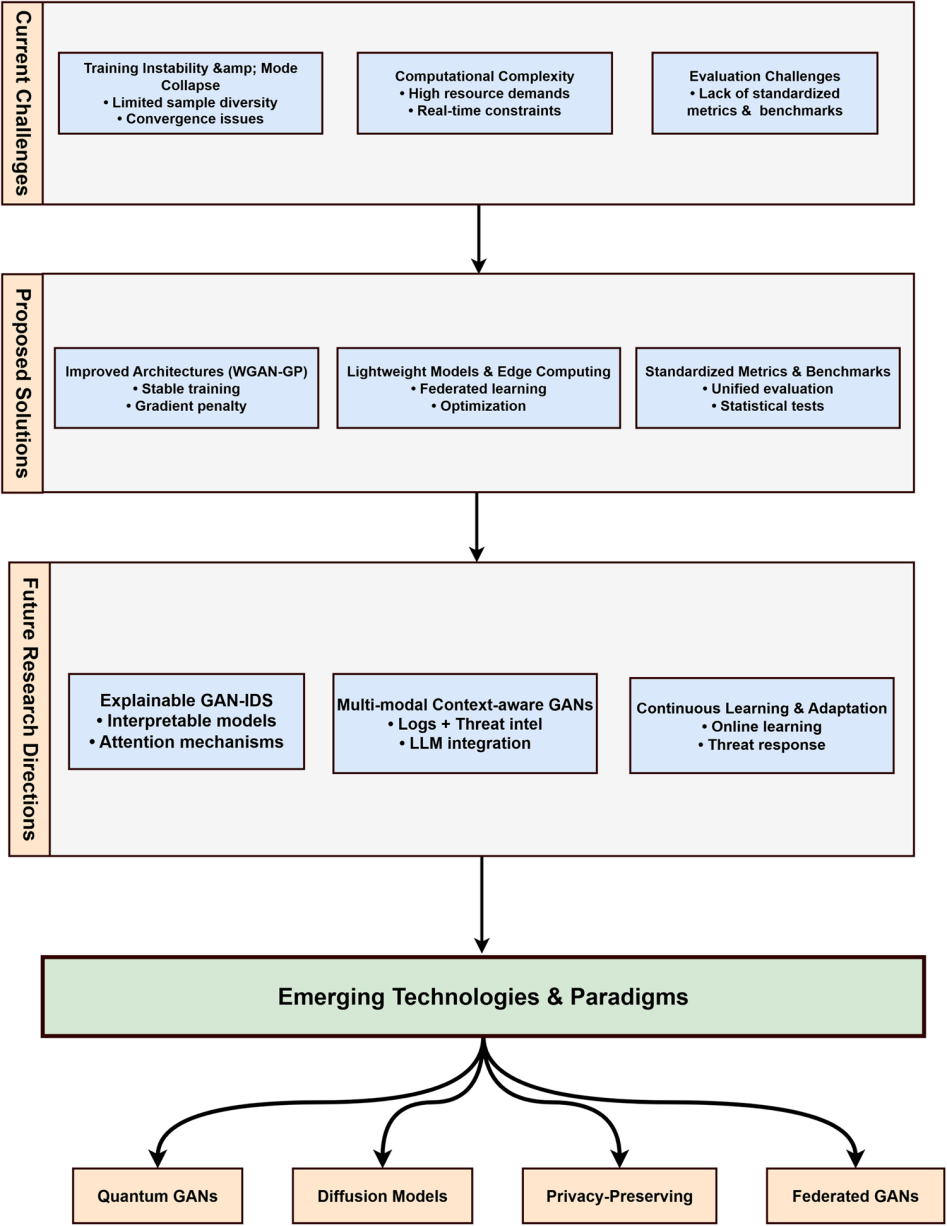
Framework illustrating the relationship between current technical challenges in GAN-based intrusion detection, existing solutions, and promising future research directions. The framework emphasizes the progression from addressing immediate technical issues to exploring advanced AI integration and interpretability

The challenges and opportunities in GAN-based intrusion detection can be systematically organized into the framework presented in Fig. 7. This framework illustrates the interconnected nature of current technical challenges, existing solutions, and future research directions.

This framework demonstrates that the development of GAN-based intrusion detection would have to be organized to develop multiple dimensions. Any technical solutions to the present challenges should be complemented with future-oriented research, which would predict future needs, particularly with the increasing complexity and prevalence of cybersecurity threats. The future advancement in this research area is the adoption of the new technologies, including large language models, quantum computing, and diffusion models.

#### Training Instability and Mode Collapse

Training instability and mode collapse remain fundamental challenges when applying GANs [1, 10, 56]. Although some variants such as WGAN?GP and modified CGANs mitigate these problems by stabilizing gradients, no architecture fully eliminates them. Further research on regularization, optimization strategies and theoretical convergence is needed.

Mode collapse presents particular challenges for intrusion detection applications, as generators that produce only limited sample varieties may fail to capture the full spectrum of attack patterns. If a GAN fails to capture the full diversity of attack patterns, it may leave blind spots in the resulting detection system. Several studies have addressed these issues through architectural innovations:

- Wasserstein GANs (WGANs) with gradient penalty have shown improved stability in multiple studies [1, 4, 5].
- Kumar and Sinha [4] incorporated regularization techniques in their WCGAN to prevent mode collapse when generating minority attack classes.
- Babu and Rao [14] modified the CGAN architecture with a Nadam optimizer to improve convergence for intrusion detection applications.

Despite these advances, this is still a challenge, especially for complex and high-dimensional network traffic data. Future research could investigate more stable training regimes or adaptive optimization techniques, or hybrid architectures which combine GANs with other generative models to mitigate these stability issues.

Few-shot learning and federated learning have become the potential solutions to the issues of data scarcity and privacy limitations. An architecture based on self-attention and meta-learning with positional encoding and refinements has been shown to be effective in few-shot intrusion detection with a 10-shot setting, achieving detection rates of 99.90% and 98.23% on the CIC-IDS2017 and CSE-CIC-IDS2018 datasets, respectively [57]. Similarly, NIDS-FGPA federated learning models, which rely on gradient-similarity aggregation to make use of Paillier homomorphic encryption to build collaborative intrusion detection system (IDS) models, achieve accuracies of 94.5% and 99.2% when using the Edge-IIoTset and CICIoT2023 benchmarks, respectively, without the raw data being exchanged; comparisons with other recent federated learning models also show improvements in performance [58]. The importance of multi-modal, privacy-conscious, and adaptive GAN-based IDS solutions can be highlighted with these developments.

#### Computational Complexity and Scalability

The computational demands of training sophisticated GANs present a significant deployment challenge. Araujo-Filho et al. [1] note that while their GAN-IDS runs about $3.8\times$ faster than LSTM-based alternatives at inference time, training remains computationally intensive. Singh et al. [16] highlight additional overhead from multi-stage pipelines (GAN training followed by Glowworm-based optimization), which may not meet real-time constraints in resource-limited environments. Future work should prioritize lightweight architectures, model compression, and distributed/federated training to reduce training and deployment costs.

For practical deployment, particularly in resource-constrained environments like IoT networks or edge devices, the computational efficiency of GAN-based systems becomes crucial. Future research directions include:

The training of advanced generative adversarial networks is a significant barrier due to the computational requirements involved in the training. Aaraujo et al. [1] noted that despite the fact that their GAN-IDS had a speed improvement of 3.8-times as compared to LSTM-based options, the initial training process was still computationally expensive. Singh et al. [16] highlighted the computational overhead that is doubled by the dual-stage training regime (consisting of a generative adversarial network and then Glowworm optimization) within their hybrid architecture as a variable that can lead to a lack of compliance with real-time detection constraints.

For practical deployment in resource-constrained environments—such as Internet of Things (IoT) systems and edge devices—the computational efficiency of GAN-based IDS pipelines is of primary importance.

- Developing lightweight GAN architectures specifically designed for resource-constrained environments
- Exploring model compression techniques to reduce the footprint of trained GAN models
- Implementing distributed or federated GAN training approaches that can leverage computational resources across multiple nodes
- Investigating transfer learning to reduce training costs by adapting pre-trained GANs to specific network environments

#### Evaluation Challenges

Evaluating GAN-based intrusion detection systems presents unique challenges that extend beyond traditional ML model assessment. A number of researchers have also observed the challenge in measuring the threat-realism of GAN-generated attack samples [10, 11]. The realism of network attack patterns is more difficult to evaluate as compared to areas such as image generation where the quality of the generated image can be assessed through visual inspection.

The measure of statistical similarity used by Kumar and Sinha [4] and Zhao et al. [5] to compare real and synthetic attack distributions could be insufficient to capture semantic aspects that are relevant to security. The dual-purpose of the GANs in the latter case, e.g., producing realistic data and enhancing levels of detection can also complicate evaluation.

Future studies would aim at the creation of special assessment systems of GAN-based security applications, such as:

- Standardized metrics for assessing the quality and diversity of synthetic attack data
- Domain-specific evaluation criteria that consider security-relevant properties of generated samples
- Benchmark datasets and protocols specifically designed for evaluating GAN-based intrusion detection
- Methods for assessing the transferability of GAN-generated attacks across different detection systems

### Research Gaps and Opportunities

Building on the technical limitations outlined in Sects. 7.1, our analysis has identified several significant research gaps that present opportunities for future work in GAN-based intrusion detection.

#### Toward Explainable GAN-Based IDS

Current GAN-based approaches often lack interpretability, functioning as “black boxes” that provide limited insight into why the discriminator raises an alert or how the generator constructs synthetic attacks. This opacity poses challenges for security professionals who need to understand and trust system outputs, particularly in high-stakes operational environments.

Across the reviewed literature, explainability is rarely treated as a first-class design objective. Future research can improve transparency for both the generator and discriminator by:

- Incorporating attention or saliency mechanisms that highlight influential features and time steps in detection decisions.
- Applying post-hoc feature attribution (e.g., SHAP-style explanations) and counterfactual analyses to discriminator outputs and downstream classifiers.
- Developing visualization techniques for GAN latent spaces and generated samples to reveal mode coverage and potential artifacts.
- Creating hybrid pipelines that combine GAN-based augmentation with inherently interpretable detectors (e.g., decision trees or rule-based models) where appropriate.

#### Multi-modal and Context-Aware GANs

Most reviewed GAN-IDS approaches operate primarily on network flow features, yet real intrusions often span multiple layers (network, host, and application) and unfold over time. Multi-modal and context-aware GANs that fuse network traffic with host logs, application telemetry, and external threat intelligence could capture richer attack behaviors and reduce false positives.

Future research could explore generative models that simultaneously model different aspects of system behavior, such as:

- Combined network traffic, system logs, and application behavior.
- Temporal context that captures attack progression and campaign structures (e.g., recurrent or transformer-based generators).
- Integration of external threat intelligence with local traffic patterns.
- Environment-aware generation that considers network topology and system configurations.

Early examples such as Mouyart et al. [30] highlight the potential of combining conditional tabular generation with other AI paradigms, but scalable multi-modal GAN designs for IDS remain largely underexplored.

#### Continuous Learning and Adaptation

The dynamic nature of cybersecurity threats demands continuous adaptation of detection systems. However, most GAN-based approaches in the literature employ static, offline training on fixed datasets, which limits their ability to respond to concept drift and newly emerging attacks. This gap creates opportunities for research on continual and online learning, including:

- Online or incremental update pipelines that retrain both the generator and discriminator as new traffic arrives.
- Techniques to prevent catastrophic forgetting (e.g., replay buffers and regularization methods such as elastic weight consolidation) when updating GAN models.
- Active learning approaches that selectively incorporate new attack patterns and prioritize labeling of informative samples.
- Reinforcement learning integration to steer generation toward difficult-to-detect behaviors and optimize update schedules based on detection outcomes.

Kamran et al. [40] began exploring this direction with a game-theoretic framework for phishing detection, but much work remains to develop robust, stable, and truly adaptive GAN-based IDS.

#### Privacy-Preserving Collaborative Defense

While several studies have explored privacy-preserving GANs for synthetic data generation [21, 48, 49], the potential for privacy-preserving collaborative defense using GANs remains underexplored. Research opportunities in this area include:

- Federated GAN training across organizations without sharing raw security data
- Differential privacy guarantees for collaborative intrusion detection
- Privacy-preserving transfer of attack knowledge between environments
- Secure multi-party computation for joint GAN-based defense mechanisms

These approaches could enable broader sharing of threat intelligence and defensive capabilities while respecting privacy concerns and regulatory requirements.

### Future Research Directions

Based on the challenges and gaps discussed in Sects. 7.1 and 7.2, we propose the following directions for future research in GAN-based intrusion detection.

#### Integration with Emerging AI Technologies

The integration of GANs with other emerging AI technologies represents a promising research direction. Several studies have begun exploring hybrid approaches:

- **GANs and Large Language Models (LLMs):** Devadiga et al. [59] introduced GLEAM, which combines GANs and LLMs for creating evasive adversarial malware. Similar ideas could be explored for intrusion detection, leveraging LLMs’ semantic understanding of attack narratives (e.g., phishing or injection payloads) alongside GANs’ ability to model numeric traffic features.
- **GANs and Reinforcement Learning:** Mouyart et al. [30] combined reinforcement learning with GAN-based synthesis for insider threat detection. Further work could explore tighter RL–GAN interactions for adaptive defense strategies and online generation objectives.
- **GANs and Quantum Computing:** Rahman et al. [23] presented an early exploration of quantum GANs (qGANs) for intrusion detection. As quantum computing advances, this direction may yield new training dynamics or computational advantages for specific subproblems.
- **GANs and Diffusion Models:** Diffusion models offer an alternative family of generative models that may alleviate some adversarial training instabilities; hybrid diffusion–GAN pipelines could be explored for high-fidelity synthesis and robustness evaluation.

While these paradigms expand the design space, they also increase system complexity and may introduce new dual-use risks. Future studies should therefore evaluate both defensive benefit and potential misuse under realistic threat models and deployment constraints.

#### Advanced Adversarial Learning for Security

The adversarial nature of GANs aligns naturally with the adversarial dynamics of cybersecurity. Future research should explore more sophisticated adversarial learning paradigms:

- **Multi-agent Adversarial Training**: Extending beyond binary generator–discriminator dynamics to multi-agent scenarios that better reflect real-world attack-defense ecosystems.
- **Adaptive Adversarial Defense**: Developing systems that continuously evolve defenses in response to emerging attack patterns, creating a moving target for adversaries.
- **Transferable Adversarial Knowledge**: Investigating how adversarial knowledge gained in one security domain can transfer to others, potentially enabling broader defensive coverage.
- **Adversarial Robustness Guarantees**: Establishing formal or empirical guarantees for the robustness of GAN-trained detection systems against specific classes of adversarial attacks.

#### Domain-Specific GAN Architectures

While many studies adapt general-purpose GAN architectures for intrusion detection, there is significant potential in developing domain-specific architectures optimized for security applications:

- **Protocol-aware GANs**: Architectures that incorporate knowledge of network protocols and their constraints to generate more realistic and semantically valid attack traffic.
- **Temporal Attack GANs**: Specialized GANs for modeling attack sequences and campaigns that unfold over time, capturing dependencies between attack stages.
- **IoT-specific GANs**: Architectures tailored to the unique characteristics of IoT traffic and constraints, building on initial work by Rahman et al. [31] and Alabsi et al. [9].
- **Infrastructure-aware GANs**: Models that consider network topology, system configurations, and organizational context when generating attack scenarios.

#### Standardization and Benchmarking

The field would benefit significantly from standardized evaluation frameworks and benchmarks specifically designed for GAN-based intrusion detection:

- **GAN-IDS Benchmark Datasets**: Creating dynamic, evolving datasets that capture modern attack patterns and network environments, addressing limitations of current static benchmarks.
- **Standardized Evaluation Metrics**: Developing agreed-upon metrics for assessing both the quality of generated attack samples and the performance of resulting detection systems.
- **Challenge Platforms**: Establishing competitive platforms where researchers can test GAN-based attack generation and detection approaches against each other in standardized environments.
- **Reproducibility Guidelines**: Creating guidelines and tools to enhance the reproducibility of GAN-based intrusion detection research, addressing a common limitation in current studies.

#### GANs for IoT and Decentralized Environments

Recent surveys observe that most GAN-based intrusion detection research assumes centrally deployed IDS and rarely evaluates models on resource-constrained Internet of Things (IoT) devices or in federated settings [11]. To broaden the scope of our survey, we include and analyze studies that deploy lightweight GAN architectures and federated IDS frameworks for IoT and edge networks. These works show that variants such as Wasserstein GANs and conditional tabular GANs can be trained on sensor data and executed on low-power devices with acceptable overhead. Future research should explore the trade-offs between model complexity, energy consumption and detection accuracy in decentralized settings, and conduct experiments on real IoT hardware or federated testbeds.

Software-defined networking (SDN) enables dynamic security policies. Integrating GAN-based IDS with SDN controllers—for example, using a dual-discriminator conditional GAN to classify SDN flows—can achieve high detection accuracy and reduce power consumption. Nayak and Bhattacharyya’s multilayered SDN security system combines MAC authentication with a dual-discriminator CGAN and reports 98.29% accuracy and a 2.05% false alarm rate while consuming less power than competing methods [46]. Including SDN-specific research in the survey highlights the relevance of GANs in programmable network environments and illustrates their applicability beyond traditional IDS deployments.

#### Standardized Evaluation Guidelines

A major obstacle to comparing GAN-based IDSs is the lack of unified datasets and performance metrics. To facilitate reproducible research, we propose that future evaluations explicitly report: (i) the dataset(s) used (e.g., NSL-KDD, UNSW-NB15, CIC-IDS2017, CSE-CIC-IDS2018, BoT-IoT or ToN_IoT); (ii) class-wise precision, recall and macro-F1-scores; (iii) per-attack recall for minority classes; and (iv) computational cost measures such as training time, inference latency and resource footprint. Researchers should also quantify how closely synthetic traffic matches real traffic using statistical similarity tests (e.g., Kolmogorov–Smirnov distance, Wasserstein distance or traffic entropy). A unified evaluation protocol will enable cross-study comparisons and meta-analyses.

#### Hyper-Parameter Tuning and Optimization Strategies

Training effective GANs requires careful selection of model architectures, loss functions, learning rates and batch sizes. Few studies systematically explore hyper-parameter optimization for GAN-IDSs. Educative’s tutorial on GAN training challenges emphasizes that hyper-parameter tuning is crucial for stable convergence [56]. We recommend reporting all relevant hyper-parameters and employing automated techniques—such as Bayesian optimization, evolutionary algorithms or meta-learning—to tune them. Open-source scripts and configuration files should accompany future work to facilitate replication and adaptation.

#### Ethical Considerations and Dual-Use Concerns

GANs have a dual-use nature: they can strengthen defenses by augmenting data, yet they can also generate realistic attack traffic that might aid adversaries. Researchers should therefore discuss the ethical implications of releasing attack generation techniques, consider controlled access to code, and evaluate potential misuse scenarios. Privacy-preserving methods such as differential privacy or federated learning can mitigate the risk of exposing sensitive data, and compliance with data-protection regulations (e.g., GDPR) is essential when sharing synthetic datasets. Incorporating ethical discussions into GAN-IDS research promotes responsible innovation.

#### Open-Source Benchmarks and Dataset Creation

The progress of GAN-based IDS research is limited by the scarcity of up-to-date and diverse intrusion datasets. We recommend that the community collaborate to develop open-source benchmark suites that capture modern attack behaviors across enterprise, cloud and IoT environments. Benchmarks should include multi-modal telemetry, scripts for GAN-based augmentation and standardized evaluation pipelines. Shared datasets and reproducible code will standardize research practices and accelerate innovation.

## Conclusion

This survey provides an in-depth analysis of generative adversarial networks (GANs) for intrusion detection. We now revisit the research objectives outlined in Section I.B and summarize how each has been addressed:

- **RQ1 (GAN architectures):** Section 4 systematically analyzes the dominant GAN architectures employed in IDS research, including Wasserstein GANs, Conditional GANs, Self-Attention GANs, and specialized multi-generator designs. We identified that WGANs and CGANs are the most commonly adopted variants due to their training stability and ability to address class imbalance, respectively. Table 1 summarizes their relative strengths and limitations.
- **RQ2 (GAN applications):** Section 5 examines five principal applications of GANs in intrusion detection: data augmentation for imbalanced datasets, adversarial training and robustness evaluation, anomaly detection, attack generation for penetration testing, and privacy-preserving synthesis. The taxonomy in Fig. 2 maps these use cases to their characteristic GAN variants and outputs.
- **RQ3 (Datasets and metrics):** Section 6 provides a comprehensive analysis of benchmark datasets (Table 2) and evaluation metrics (Table 3) used in GAN-based IDS research. We identified NSL-KDD, UNSW-NB15, and CIC-IDS2017 as the most widely used datasets, while highlighting the growing importance of IoT-specific datasets such as BoT-IoT and CICIoT2023.
- **RQ4 (Challenges and future directions):** Section 7 identifies key technical challenges including training instability, mode collapse, computational complexity, and evaluation difficulties. We proposed future research directions spanning explainable GAN-IDS, multi-modal approaches, continuous learning, and integration with emerging AI paradigms such as LLMs, quantum computing, and diffusion models.
- **RQ5 (Dual-use nature):** Throughout the survey, particularly in Sects. 5.2 and 5.4, we examined how GANs serve both defensive purposes (data augmentation, adversarial training) and offensive applications (evasion attack generation, penetration testing). This dual-use nature underscores the importance of responsible disclosure and ethical considerations discussed in Sect. 7.

By addressing these research questions, this survey provides a comprehensive and forward-looking reference for practitioners and researchers developing robust, privacy-preserving, and adaptive GAN-based intrusion detection systems.
